# Draft Genome Sequences of Four Bacterial Strains Isolated from Sediment of the South China Sea

**DOI:** 10.1128/mra.00191-22

**Published:** 2022-04-13

**Authors:** Sidra Erum Ishaq, Tariq Ahmad, Jialin Hou, Lewen Liang, Yinzhao Wang, Fengping Wang

**Affiliations:** a State Key Laboratory of Microbial Metabolism, Joint International Research Laboratory of Metabolic & Developmental Sciences, School of Life Sciences and Biotechnology, Shanghai Jiao Tong University, Shanghai, China; b School of Oceanography, Shanghai Jiao Tong University, Shanghai, China; University of Southern California

## Abstract

Here, we report the draft genome sequences of four bacterial isolates from sediment of the South China Sea. Three of the isolates belong to the class *Alphaproteobacteria* and encode complete SoxXAYZBCD gene clusters, related to thiosulfate oxidation, while one isolate belongs to the class *Opitutae* and possesses a total of 397 carbohydrate active enzymes (CAZymes), related to predicted polysaccharide degradation.

## ANNOUNCEMENT

Marine ecosystems are a vast habitat for diverse metabolically active groups of microbes, including sulfur-oxidizing and polysaccharide-degrading bacteria (1, 2). In the context of screening and isolating novel and uncultured metabolically active bacterial strains from marine settings in our laboratory (3–5), four strains have been isolated from sediment samples of the South China Sea (global positioning system [GPS] coordinates, 115.830°E, 18.813°N). The sediment cores were collected using a multicorer, sliced into 2-cm pieces onboard as soon as possible, placed into Nasco sampling bags, and preserved at 4°C.

Isolation of the strains was achieved by serial dilutions of a 1% sediment sample in artificial sea water (6). Dilutions of 10^–6^ and 10^–7^ were inoculated aerobically onto the isolation agar media listed in Table 1 and incubated at 25°C for several weeks. The observed selected colonies were purified on marine R2A agar plates; after successful purification of the colonies, PCR and 16S rRNA gene sequencing and analysis was performed (7). The strains were preserved at −80°C using the glycerol and skimmed milk method (8, 9).

**TABLE 1:** Genome assembly details and statistics

Characteristic	Data for strain:			
	LMO-S08	LMO-JJ12	LMO-JJ14	LMO-M01
Isolation agar medium	R2A^*a*^	M11	R2A	R2A
SRA accession no. (raw data)	SRR17583017	SRR17583015	SRR17583014	SRR17583016
No. of reads	8,487,834	13,462,948	10,486,140	12,487,714
Read length (bp)	1,273,175,100	2,019,442,200	1,572,921,000	1,958,064,900
Genome size (bp)	3,976,845	43,469,495	3,875,047	5,736,558
Coverage (×)	99.2	99.2	97.6	83.9
No. of contigs	9	10	15	48
No. of CDSs^*b*^	3,837	4,205	3,693	4,522
GC content (%)	64.46	58.99	58.82	61.34
*N*_50_ (bp)	1,071,250	2,501,644	2,130,079	216,446
No. of tRNAs	46	42	45	53
Water/sediment depth (m/cm)	3,219.7/440–460	1,896.9/60–160	1,896.9/60–160	1,896.9/60–160
GenBank accession no.	JAKGBY010000000	JAKGBX010000000	JAKGCX010000000	JAKGCW000000000
Taxonomy				
Kingdom	Bacteria	Bacteria	Bacteria	Bacteria
Phylum	Proteobacteria	Proteobacteria	Proteobacteria	Verrucomicrobia
Class	Alphaproteobacteria	Alphaproteobacteria	Alphaproteobacteria	Opitutae
Order	Rhodospirillales	Rhodobacterales	Rhodospirillales	Opitutales
Family	Thalassospiraceae	Rhodobacteraceae	Thalassospiraceae	Opitutaceae
Genus	Magnetospira	Octadecabacter	Varunaivibrio	Cephaloticoccus
Closest cultured strain	Magnetospira thiophila MMS^T^	Octadecabacter antarcticus 307^T^	Varunaivibrio sulfuroxidans TC8^T^	Cephaloticoccus primus CAG34^T^
16S rRNA similarity (%)	90.57	96.47	91.03	92.50
ANI^*c*^	76.7	76.69	76.29	76.57

a R2A, Reasoner’s 2A.

b CDSs, coding DNA sequences.

c ANI, average nucleotide identity.

For DNA extraction, all four strains were grown in Marine R2A broth medium for 5 days at 25°C on a rotary shaker (150 rpm). DNA was extracted using a DNeasy PowerSoil kit (Qiagen, Germany) according to the manufacturer’s instructions. The DNA concentration was quantified by the absorbance at 260 nm using a NanoDrop spectrophotometer. A library was prepared following the workflow of the TruSeq DNA library preparation kit (Illumina), then sequenced to generate 150-bp paired-end reads on the Illumina HiSeq 2500 platform.

Raw data for each strain was generated from their respective raw reads, which were assembled and quality checked using SPAdes v3.11.1 (10) and FastQC v0.11.8 (11), respectively, after filtering the low-quality reads (Q scores, ≤5) and adaptor sequences using Trimmomatic v0.38 (12). The assembled contigs were retrieved as draft genomes, with the respective size and GC content for each strain as shown in Table 1. The final assemblies were annotated using the NCBI Prokaryotic Genome Annotation Pipeline (PGAP) within the best-placed reference protein set and GeneMarkS-2+ v5.3 software with annotation (13). The total number of open-reading frames (ORFs) was predicted using PGAP within the draft genomes, which were further annotated using KEGG, COG, Pfam, and the NCBI nonredundant database with DRAM v1.2.0 (14). The noncoding RNA genes were predicted using tRNAscan-SE v2.0.9 (15); the draft genome of each strain has one 16S rRNA gene, one 5S rRNA gene, and one 23S rRNA gene, while the number of tRNA genes varies (listed in Table 1). Default parameters were used for all software unless otherwise specified. All genome sequence-related data are summarized in Table 1. Three of these strains, LMO-S08, LMO-JJ12, and LMO-JJ14, belong to the class *Alphaproteobacteria*, which encodes the complete Sox pathway for thiosulfate oxidation, while strain LMO-M01, belonging to the class *Opitutae* (phylum *Verrucomicrobia*), has 397 carbohydrate-active enzymes (CAZymes), including 37 carbohydrate-binding modules, 83 carbohydrate esterases, 139 glycoside hydrolases, 107 glycosyl transferases, 18 polysaccharide lyases, and 13 auxiliary activities.

### Data availability.

The assembled genome sequences and raw data have been deposited at GenBank under the accession numbers listed in Table 1.

## References

[1] Jorgensen BB, Findlay AJ, Pellerin A. 2019. The biogeochemical sulfur cycle of marine sediments. Front Microbiol 10:849. doi:10.3389/fmicb.2019.00849. 31105660 PMC6492693

[2] Sun H, Gao L, Xue C, Mao X. 2020. Marine polysaccharide degrading enzymes: status and prospects. Compr Rev Food Sci Food Saf 19:2767–2796. doi:10.1111/1541-4337.12630. 33337030

[3] Wang Y, Kamagata Y, Li M, Han F, Wang F, Xiao X. 2021. New approaches for archaeal genome-guided cultivation. Sci China Earth Sci 64:1658–1673. doi:10.1007/s11430-020-9793-5.

[4] Hu H, Natarajan VP, Wang F. 2021. Towards enriching and isolation of uncultivated archaea from marine sediments using a refined combination of conventional microbial cultivation methods. Mar Life Sci Technol 3:231–242. doi:10.1007/s42995-021-00092-0. PMC10077295 37073339

[5] Yu T, Wu W, Liang W, Lever MA, Hinrichs KU, Wang F. 2018. Growth of sedimentary Bathyarchaeota on lignin as an energy source. Proc Natl Acad Sci USA 115:6022–6027. doi:10.1073/pnas.1718854115. 29773709 PMC6003339

[6] Zhang J, Liu R, Xi S, Cai R, Zhang X, Sun C. 2020. A novel thiosulfate oxidation pathway provides a new clue about the formation of zero-valent sulfur in deep sea. ISME J 14:2261–2274. doi:10.1038/s41396-020-0684-5. 32457501 PMC7608252

[7] Bergkessel M, Guthrie C. 2013. Colony PCR, p 299–309. *In* Lorsch J (ed), Methods in enzymology, vol 529. Academic Press, San Diego, CA. doi:10.1016/B978-0-12-418687-3.00025-2. 24011056

[8] Kaeberlein T, Lewis K, Epstein SS. 2002. Isolating “uncultivable” microorganisms in pure culture in a simulated natural environment. Science 296:1127–1129. doi:10.1126/science.1070633. 12004133

[9] Chaudhary DK, Khulan A, Kim J. 2019. Development of a novel cultivation technique for uncultured soil bacteria. Sci Rep 9:6666. doi:10.1038/s41598-019-43182-x. 31040339 PMC6491550

[10] Bankevich A, Nurk S, Antipov D, Gurevich AA, Dvorkin M, Kulikov AS, Lesin VM, Nikolenko SI, Pham S, Prjibelski AD, Pyshkin AV, Sirotkin AV, Vyahhi N, Tesler G, Alekseyev MA, Pevzner PA. 2012. SPAdes: a new genome assembly algorithm and its applications to single-cell sequencing. J Comput Biol 19:455–477. doi:10.1089/cmb.2012.0021. 22506599 PMC3342519

[11] Andrews S. 2017. FastQC: a quality control tool for high throughput sequence data. https://www.bioinformatics.babraham.ac.uk/projects/fastqc/.

[12] Bolger AM, Lohse M, Usadel B. 2014. Trimmomatic: a flexible trimmer for Illumina sequence data. Bioinformatics 30:2114–2120. doi:10.1093/bioinformatics/btu170. 24695404 PMC4103590

[13] Tatusova T, DiCuccio M, Badretdin A, Chetvernin V, Nawrocki EP, Zaslavsky L, Lomsadze A, Pruitt KD, Borodovsky M, Ostell J. 2016. NCBI Prokaryotic Genome Annotation Pipeline. Nucleic Acids Res 44:6614–6624. doi:10.1093/nar/gkw569. 27342282 PMC5001611

[14] Shaffer M, Borton MA, McGivern BB, Zayed AA, La Rosa SL, Solden LM, Liu P, Narrowe AB, Rodríguez-Ramos J, Bolduc B, Gazitúa MC, Daly RA, Smith GJ, Vik DR, Pope PB, Sullivan MB, Roux S, Wrighton KC. 2020. DRAM for distilling microbial metabolism to automate the curation of microbiome function. Nucleic Acids Res 48:8883–8900. doi:10.1093/nar/gkaa621. 32766782 PMC7498326

[15] Lowe TM, Eddy SR. 1997. tRNAscan-SE: a program for improved detection of transfer RNA genes in genomic sequence. Nucleic Acids Res 25:955–964. doi:10.1093/nar/25.5.955. 9023104 PMC146525

